# Effect of Inducible BMP-7 Expression on the Osteogenic Differentiation of Human Dental Pulp Stem Cells

**DOI:** 10.3390/ijms22126182

**Published:** 2021-06-08

**Authors:** Ferenc Tóth, József Tőzsér, Csaba Hegedűs

**Affiliations:** 1 Department of Biomaterials and Prosthetic Dentistry, Faculty of Dentistry, University of Debrecen, 4032 Debrecen, Hungary; hegedus.csaba.prof@dental.unideb.hu; 2 Department of Biochemistry and Molecular Biology, Faculty of Medicine, University of Debrecen, 4032 Debrecen, Hungary; tozser@med.unideb.hu

**Keywords:** bone morphogenetic protein 7, dental stem cells, gene therapy, regenerative medicine, osteogenic differentiation, tet-on

## Abstract

BMP-7 has shown inductive potential for in vitro osteogenic differentiation of mesenchymal stem cells, which are an ideal resource for regenerative medicine. Externally applied, recombinant BMP-7 was able to induce the osteogenic differentiation of DPSCs but based on our previous results with BMP-2, we aimed to study the effect of the tetracyclin-inducible BMP-7 expression on these cells. DPSC, mock, and DPSC-BMP-7 cell lines were cultured in the presence or absence of doxycycline, then alkaline phosphatase (ALP) activity, mineralization, and mRNA levels of different osteogenic marker genes were measured. In the DPSC-BMP-7 cell line, the level of BMP-7 mRNA significantly increased in the media supplemented with doxycycline, however, the expression of Runx2 and noggin genes was upregulated only after 21 days of incubation in the osteogenic medium with doxycycline. Moreover, while the examination of ALP activity showed reduced activity in the control medium containing doxycycline, the accumulation of minerals remained unchanged in the cultures. We have found that the induced BMP-7 expression failed to induce osteogenic differentiation of DPSCs. We propose three different mechanisms that may worth investigating for the engineering of expression systems that can be used for the induction of differentiation of mesenchymal stem cells.

## 1. Introduction

Regenerative medicine is an interdisciplinary and rapidly developing field of industry and medicine since the last decades [1]. Besides tissue engineering, which provides the appropriate niche (scaffold materials) for the differentiation of the tissue-specific cell phenotypes, the application of growth factors and embryonal or adult stem cells is also required for successful treatment of any tissue types [1,2,3,4,5].

A significant number of adult mesenchymal stem cells with various differentiation and proliferation potential derive from the oral cavity [6,7]. These stem cells are mainly tooth-related cells derived from different tissues, such as dental pulp stem cells (DPSCs), stem cells from apical papilla (SCAPs), periodontal ligament stem cells (PDLSCs), dental follicle progenitor cells (DFPCs), tooth germ stem cells (TGSCs), gingiva-derived mesenchymal stem cells (GMSCs), alveolar bone marrow mesenchymal stem cells (ABMSCs), and stem cells from human exfoliated deciduous teeth (SHEDs). The simplicity of tissue harvest, the high proliferative activity, differentiation capabilities, and immunomodulatory properties of these cells offer several opportunities for application in regenerative medicine. Moreover, most of these cells were already proved to be useful for the regeneration of bone and other tissue types [8,9,10,11,12].

Human dental pulp stem cells (DPSCs) are adult, mesenchymal stem cells isolated from third molars [13,14]. Besides their high proliferative potential and ease of cryopreservation [15], their differentiation potential [16,17] and the ability to regulate the function of other cell types [18,19] makes them very appropriate cells for the regeneration of several tissue types [8,20,21] including bone and tooth [10,22,23,24].

The regulated expression of specific differentiation factors is quite often critical for the successful application of stem cells in regenerative medicine [3,4,25]. The lack of control in case of the expression (or local application of the recombinant protein) of these factors can lead to tumor formation [26,27], or as a result of inflammation caused by this phenomenon edema formation and tissue damage may happen in the application environment [28,29], or even have an adverse effect on the regeneration of the bone tissue [30]. The tetracyclin induced (tet-on) gene expression systems [31], which can stop the expression of the transgene by the withdrawal of the inducing agent, are widely used systems for this purpose and are successfully applied for the expression of genes related to osteogenic differentiation and osteogenesis [17,32,33,34].

Bone morphogenetic protein 7 (BMP-7) is a member of the transforming growth factor-β superfamily, many of which members have important functions in skeletal development, regeneration, and repair. BMP-7 has an important role in the development of several tissue types including teeth and bone [35,36] and is known to act as an inducer of osteoblast/odontoblast differentiation in vitro and in vivo [37,38,39,40,41,42]. It was also extensively used for clinical application in the treatment of long bone nonunions and spinal fusion [43,44]. However, several concerns have been raised regarding the dose-dependent side effects of the application of the protein [28,29].

Although BMP-7 was proved to be an effective inducer of the odontogenic differentiation of DPSCs [30] in vitro, the previously raised concerns regarding the dose-dependent side effects of the clinically used recombinant protein, and the experienced difficulties with the delivery of BMPs by various carriers [45,46,47] motivated our search for a more viable alternative that can promote the osteogenic differentiation of the cells without the aforementioned side effects caused by the uncontrolled use of differentiation factors. Therefore, the present study aimed to investigate the effect of inducible BMP-7 gene expression on the osteogenic differentiation of DPSCs. For this purpose, we have used a DPSC cell line capable of tet-inducible BMP-7 expression [48] with the application of the previously described vector system [32,49]. 

## 2. Results

### 2.1. Doxycycline-Regulated Gene Expression

pTet-IRES-EGFP-BMP-7 and pLenti CMV rtTA3 Blast viral pseudoparticles were used to transduce a DPSC cell line, then the GFP expression of the untreated and doxycycline-treated cells were examined by fluorescent microscopy. Also, the BMP-7 expression in the cell lysates of the DPSC-BMP7 cell line, cultured in CM, CM+, OM, and OM+ was examined by western blot analysis, and the BMP-7 content of the cell supernatants was investigated by BMP-7 ELISA. 

**Figure 1:**
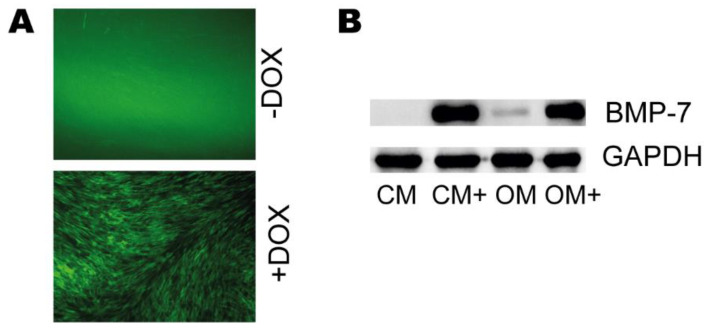
(**A**) Representative images of the DPSC-BMP-7 cell line incubated in the presence (DOX) or absence (−DOX) of doxycycline. (**B**) Western blot analysis of DPSC-BMP7 cell lysates.

Our results show that the addition of doxycycline was successfully induced the GFP expression of the DPSC-BMP-7 cells (Figure 1A). 

The examination of the cell lysates has revealed no or a very little amount of BMP-7 precursor protein in the cells when grown without doxycycline (CM and OM) (Figure 1B), while a significant amount of the protein was detected in the presence of the inducing agent (CM+ and OM+).

The cell supernatants demonstrated differences in the BMP-7 content amongst the cells grown in different media using ELISA, similar to the results observed with the western blot analysis. Very low amount of BMP-7 found in the supernatants of the cells (Table 1) that were incubated in the control or osteogenic medium without doxycycline (CM and OM) at every day that was investigated. While in the presence of doxycycline, both media (CM+ and OM+) contained significantly higher amounts that were grown even higher with the extension of the incubation period.

### 2.2. Quantitative Real-Time PCR Assays

The expression of BMP-7, noggin, and Runx2 genes was examined after 1 and 3 weeks of culture in the presence (CM+ and OM+) or absence (CM and OM) of 100 ng/mL doxycycline.

**Figure 2:**
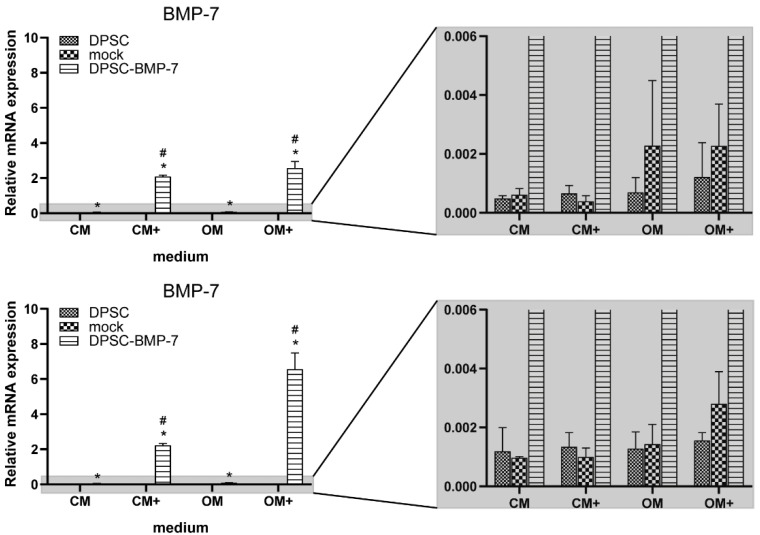
The expression level of BMP-7 mRNA in the case of the indicated cell lines. After 1 week (above) and 3 weeks (below) of induction in different media (mean ± SD; *n* = 3). *, *p* ˂ 0.05 vs. control in the same media, #, *p* ˂ 0.05 vs. untreated (CM or OM respectively) group of the same cell line.

After 1 week, a significant increase was observed in the gene expression of BMP-7 in the DPSC-BMP-7 cell line grown in any media compared to that of the control (Figure 2). This expression in DPSC-BMP-7 grown in CM+ and OM+ was significantly higher when compared to CM and OM, respectively. The same effect was observed after 21 days of incubation, however, the expression level in OM+ was more than 2 times higher than it was observed at the previous time point.

**Figure 3:**
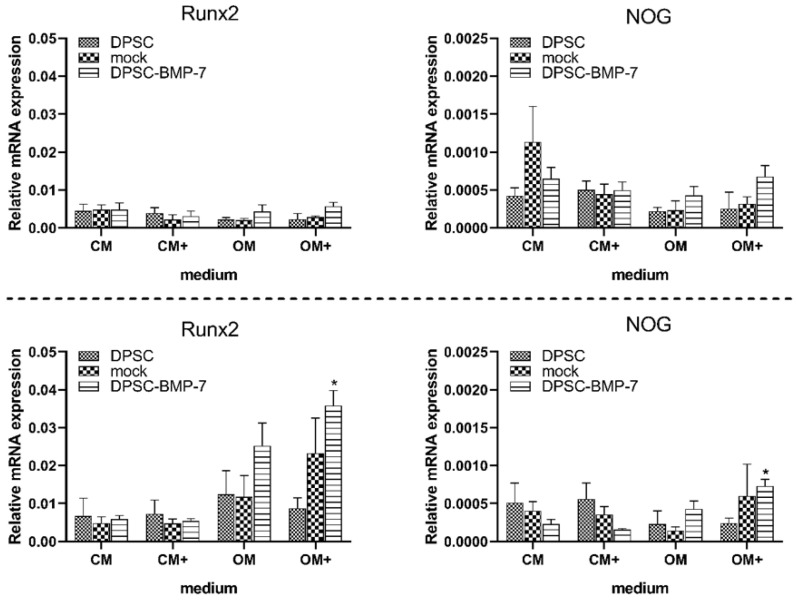
Expression level of Runx2 and noggin (NOG) mRNA in case of the indicated cell lines. After 1 week (above) and 3 weeks (below) of induction in different media (mean ± SD; *n* = 3). *, *p* ˂ 0.05 vs. control (DPSC) in the same media, #, *p* ˂ 0.05 vs. untreated (CM or OM respectively) group of the same cell line.

The gene expression of Runx2 did not show any significant alterations in the mock or DPSC-BMP-7 cells (Figure 3) compared to the control after 7 days, but it was significantly higher in DPSC-BMP-7 in OM+ after 21 days of incubation. However, this increased gene expression was proved to be not significantly different when compared to the DPSC-BMP-7 cultured in OM, suggesting, that it is not in part due to the highly elevated BMP-7 gene expression. Also, the addition of doxycycline (CM+ and OM+) could not initiate any change in the Runx2 gene expression in any of the examined cell lines or time-points when compared to CM or OM, respectively. 

Similar trends were observed in the case of noggin gene expression in all the examined cell lines and media, suggesting that unlike in the case of the BMP-2 overexpression [21] this inhibitor has no significant role in the failure of BMP-7 induced osteogenic differentiation.

### 2.3. Alkaline Phosphatase Assay

**Figure 4:**
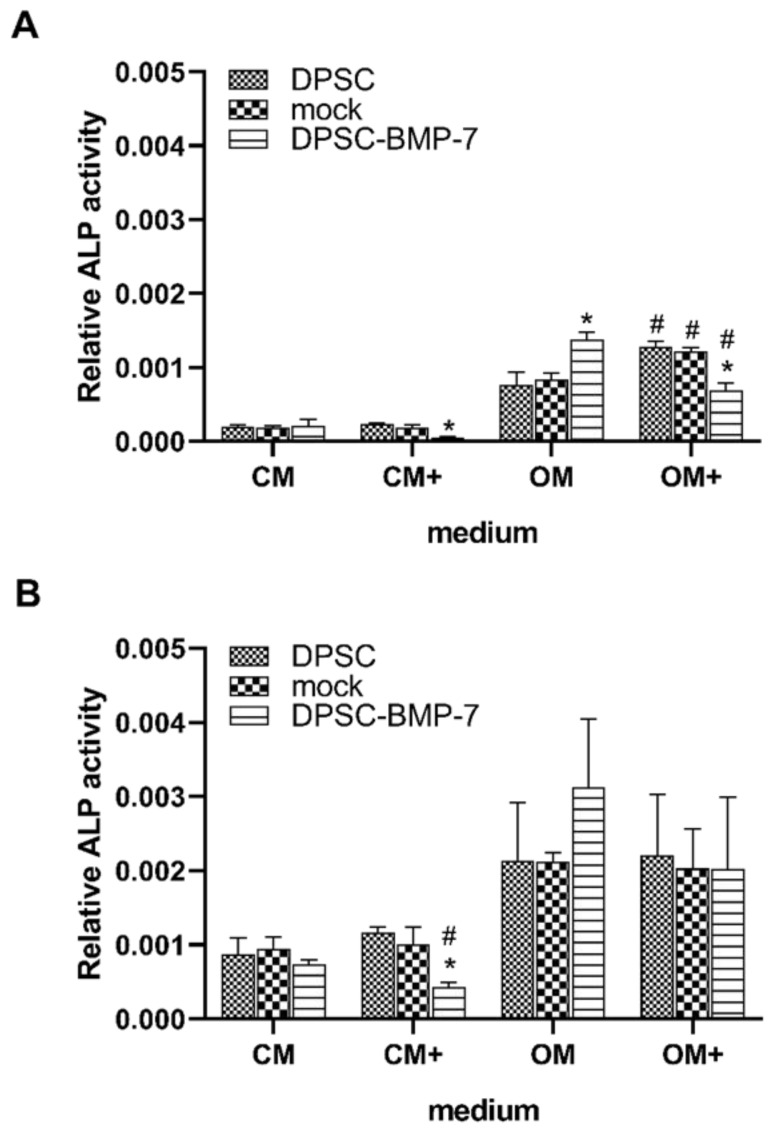
ALP activity of the indicated cell lines. After 1 week (**A**) and 3 weeks (**B**) of induction in different media. *, *p* ˂ 0.05 vs. control (DPSC) in the same media, #, *p* ˂ 0.05 vs. untreated (CM or OM respectively) group of the same cell line.

The activity of the alkaline phosphatase enzyme was examined after 1 week and 3 weeks of culture. After 1 week of incubation, the ALP activity of the DPSC-BMP-7 was significantly reduced in CM+ and OM+ (Figure 4A), while increased in OM compared to the control cell line, and in the case of OM+ to the DPSC-BMP-7 grown in OM as well. Besides, the addition of doxycycline significantly increased the ALP activity of the DPSC and mock cell line in OM+ compared to that of OM.

After 21 days, most of the alterations observed earlier were restored, only the DPSC-BMP-7 in CM+ showed a reduction in its ALP activity (Figure 4B) compared to that of the control and the activity measured in CM.

### 2.4. Mineralization Assay

**Figure 5:**
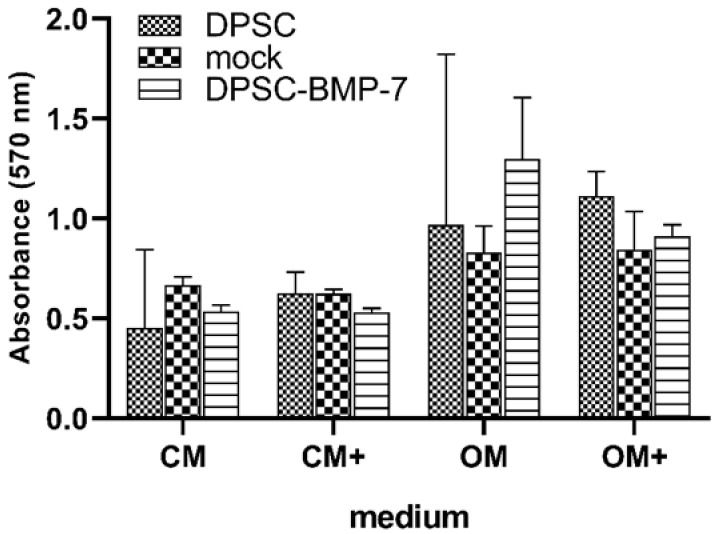
Mineralization of the indicated cell lines grown for 3 weeks in different media. Cells were stained and alizarin red levels taken were quantified with the use of cetylpyridinium chloride at 570 nm. *, *p* ˂ 0.05 vs. control (DPSC) in the same media, #, *p* ˂ 0.05 vs. untreated (CM or OM respectively) group of the same cell line.

Mineralization was measured in all samples after 3 weeks of incubation. As it is shown in Figure 5, there is no significant difference between the mineralization of the mock or DPSC-BMP-2 cell lines compared to the control, and no significant alteration was found in any of the cell lines as the possible effect of the addition of doxycycline compared to CM and OM, respectively. It seems that similar to the other osteogenic markers examined in this study, the induced BMP-7 expression was not able to increase the calcium deposition of the DPSC-BMP-7 cell line.

## 3. Discussion

Among the various stem cell types, mesenchymal stem cells of the oral cavity provide a very attractive source for cell-based bone and tooth regeneration. These cells are not only easy to handle in vitro, but also have high proliferative activity, show strong osteogenesis capacity, and have immunomodulatory properties that make them ideal candidates for tissue regeneration purposes [6,7,50].

In this study, we have examined a dental pulp stem cell line that is capable of the inducible overexpression of the osteogenic differentiation inducer BMP-7 protein. We have found that unlike external BMP-7 addition [41], the induction of BMP-7 expression in the DPSC-BMP-7 cell line failed to induce osteogenic differentiation. 

However, after 3 days of incubation, the supernatant of the induced DPSC-BMP-7 cells contained an ideal amount of BMP-7, after 2 days only OM+, while after 1 day neither of the induced media contained enough BMP-7 for the induction of osteogenic differentiation [41]. We presume, that after reaching the maximum concentration it remains steady by the cooperation of the continuous protein expression and the work of proteolytic enzymes. While, in the case of the externally applied BMP-7 a significant amount of protein will be degraded by the proteolytic enzymes, and without supply, it becomes less throughout the incubation period.

BMPs and BMP receptors control the differentiation of mesenchymal stem cells [38,39,51,52] in very complex cooperation with several other pathways and transcription factors [53] such as Wnt, Notch, fibroblast growth factor (FGF), or sonic hedgehog (Shh) and players of these signaling pathways [54]. These pathways can be also altered by several factors that may affect the differentiation of mesenchymal stem cells [55,56,57]. The evaluation of our data also revealed, that after 1 week of incubation, the induced BMP-7 expression negatively affects the ALP activity of the DPSC-BMP-7 cell line both in CM+ and OM+ and this effect is prolonged to 3 weeks in CM+. As the addition of doxycycline did not affect the ALP activity of the other cell lines, doxycycline can be excluded as the possible agent causing this phenomenon. These results and the examination of the other differentiation markers such as the mineralization of the cell line also support the inability of the system to induce osteogenic differentiation, which can be inhibited by many of the inhibitors of the aforementioned pathways. 

The evaluation of the data from the quantitative real-time PCR assays also revealed, that while BMP-7 expression was significantly higher in the DPSC-BMP-7 cell line when cultured in the presence of doxycycline both in CM and OM, this increase did not significantly alter the expression of the differentiation marker Runx2 or the BMP antagonist noggin. The constant expression of noggin suggests that this recently identified inhibitor of the effect of induced BMP-2 expression in DPSCs [32] has no role in the failure of the induction of early differentiation of DPSCs into the osteoblastic lineage. Whereas other inhibitors that may alter the effect of BMP signaling were not investigated, we presume that a not yet identified player of the osteoblastic differentiation regulatory pathways might be behind this phenomenon, which has been activated as a reaction for the increased BMP-7 expression in the DPSC-BMP-7 cell line.

Nevertheless, recent studies demonstrated, that many of the positive effects exerted by the mesenchymal stem cells, is due to their secreted exosomes [58,59,60] that are important tools for intercellular communication, that provide new therapeutic alternatives for tissue engineering and regeneration. These exosomes are extracellular vesicles containing different signaling molecules, including mRNA and proteins that can mediate the osteogenic differentiation of other cells [61]. The effect of the exosomes of several stem cells derived from the oral cavity on osteogenesis has been also demonstrated recently [62,63,64]. The contents of these secreted vesicles vary with cell types [63] and cellular states, hence may have different effects on the differentiation of the target cells [50]. Based on these earlier observations, we also hypothesize that the forced expression of BMP-7 is altering the composition of the exosomes and via this paracrine activity BMP-7 cannot exert the expected positive effect on cell differentiation.

Although our results showed that the inducible BMP-7 expression failed to induce the osteogenic differentiation of DPSCs in vitro, it was designed to investigate the possible effect of the inducible BMP-7 expression, therefore, in the light of the results, this study has some limitations. (1) We have not used additional experimental controls to compare the details of the effects of the externally applied protein and our expression system, which may give appropriate information about the factors that affect the different results from the two approaches. (2) The study focused on the investigation of the BMP antagonist noggin, however, there are more inhibitors of the BMP signaling pathway that may be responsible for the failure of the induction of osteogenic differentiation by the inducible BMP-7 expression. (3) Exosome composition of the cell lines involved in the study was not examined. 

We believe that our findings and/or further investigations, designed to address the aforementioned limitations of this study may give useful information to understand the mechanisms behind the function of the tet-inducible gene expression system or find alternatives that may effectively support the in vivo therapeutic application of mesenchymal stem cells and can be used for the induction of differentiation in stem cells of different origin, including the mesenchymal stem cells of the oral cavity.

## 4. Materials and Methods

### 4.1. Construction of pTet-IRES-EGFP-BMP-7 Plasmid

The cDNA segment coding BMP-7 mRNA (Genscript, Piscataway, NJ, USA) was ligated into the pTet-IRES-EGFP plasmid [36] at the BamHI restriction site. The plasmid pTet-IRES-EGFP was a gift from Maria Lung (Addgene plasmid # 64238). The ligation mixture was then transformed as described earlier [50]. The resultant recombination vector was referred to as pTet-IRES-EGFP-BMP-7.

### 4.2. Cell Culture

DPSC, mock, and DPSC-BMP-7 cell lines were cultured in DMEM F12 supplemented with 10% fetal bovine serum, 1% Glutamax, and 1% Antibiotic-Antimycotic (All from Thermo Fisher Scientific, Waltham, MA, USA). This medium is referred to in the text as a control medium (CM). Osteoinductive medium (OM) was prepared by supplementing CM with 10 mM β-glycerophosphate (Sigma Aldrich, St Louis, MO, USA), 50 µg/mL ascorbic acid (Sigma Aldrich, St Louis, MO, USA), 0.1 µM dexamethasone (Sigma Aldrich, St Louis, MO, USA), and 50 nM vitamin D3 (Sigma Aldrich, St Louis, MO, USA). Media for inducing transgene expression were supplemented by 100 ng/mL doxycycline (Sigma Aldrich, St Louis, MO, USA) (indicated as CM+ and OM+ respectively).

The cell lines were plated at a density of 4 × 10^4^ cells/mL in 24 well plates. The plates were cultured at 37 °C in 5% CO_2_. After 24 h, the medium was replaced with different media.

### 4.3. Lentivirus Preparation and Transduction

The viral particle production, subsequent transduction, antibiotic selection, and examination of the transduction efficiency were performed as previously described [21] with the use of the pTet-IRES-EGFP-BMP-7 (DPSC-BMP-7) or pTet-IRES-EGFP (mock) plasmid and viral particles. Briefly, viral particles were produced by transient transfection of 293FT at approximately 70% confluence and the following plasmids were used in T75 flasks: 8 µg pTet-IRES-EGFP, pTet-IRES-EGFP-BMP-7, or pLenti CMV rtTA3 Blast (for reverse tetracycline-controlled transactivator 3 expression), 6 µg psPAX2, 2 µg pMD2.G, and 13 µg salmon sperm DNA (Sigma Aldrich, St Louis, MO, USA). The medium was replaced after 6 h and the conditioned media containing virus particles were collected after 3 days, clarified by centrifugation, and was filtered through a 0.45 µm polyvinylidene fluoride (PVDF) filter (Millipore, Billerica, MA, USA), then stored in −70 °C.

For transduction, 500 µL pLenti CMV rtTA3 Blast (w756-1) and 500 µL pTet-IRES-EGFP-BMP-2 or pTet-IRES-EGFP (mock) virus particles and 1 mL fresh medium containing 8 µg/mL polybrene (Sigma Aldrich, St Louis, MO, USA) were mixed with and this mixture was used for transduction in 6-well plates containing DPSC cells at 50% confluency. After overnight incubation, the medium was changed to CM. Medium containing 5 µg/mL blasticidin (Thermo Fisher Scientific, Waltham, MA, USA) was added to the cells at passage one to perform an antibiotic selection of the cells transduced by the viral particles containing pLenti CMV rtTA3 Blast. After that, GFP expression was induced by the addition of 100 ng/mL doxycycline to identify successfully co-transduced cells.

### 4.4. Western Blot Analysis

10^5^ cells were seeded to Petri dishes and cultured for 6 days in CM or OM in the presence or absence of 100 ng/mL doxycycline. After 6 days, protein transport from the endoplasmic reticulum to the Golgi apparatus was inhibited by the addition of 10 µg/mL Brefeldin A (Tocris Bioscience, Tocris, United Kingdom). 24 h later, the cells were lysed with radioimmunoprecipitation assay (RIPA) buffer. The protein concentration was determined by Pierce BCA Protein Assay (Thermo Fisher Scientific, Waltham, MA, USA), then 10 µg lysates from each sample were resolved by 14% SDS-PAGE analyzed by western blot using BMP-7 (6E5D12) antibody (Santa Cruz Biotechnology, Dallas, TX, USA) and Anti-GAPDH Antibody (Lifespan Biosciences, Seattle, WA, USA) respectively.

### 4.5. BMP-7 ELISA

4 × 10^4^ cells were plated into 24-well plates then let to attach overnight. The other day medium was replaced with CM, CM+, OM, or OM+ respectively. After 1, 2, or 3 days, the supernatants of the cells were collected and stored at −20 °C until ELISA measurement.

Human BMP7 ELISA Kit (Proteintech Group, Rosemont, IL, USA) was used to assess the level of BMP-7 in the cell supernatants.

### 4.6. Real-Time Quantitative PCR

After 1 and 3 weeks, the total RNA of the cells was isolated with Quick-RNA MiniPrep kit from Zymo Research (Irvine, CA, USA), and cDNA was synthesized from 1 µg total RNA by using High-Capacity cDNA Reverse Transcription Kit from Applied Biosystems. All reactions were performed in triplicates and analyzed. Real-time quantitative PCR was performed by the incubation of 5x HOT FIREPol Probe qPCR Mix Plus (no ROX) from Solis BioDyne (Tartu, Estonia) Applied Biosystems Taqman Assays: BMP-7 (Hs00233476_m1), Noggin (Hs00271352_s1), Runx2 (Hs00231692_m1) (all from Thermo Fisher Scientific, Waltham, MA, USA). The expression level was normalized by GAPDH (glycerol-aldehyde-3-phosphate dehydrogenase, Hs02758991_g1) expression.

### 4.7. Detection of Alkaline Phosphatase (ALP) Activity

After 1 and 3 weeks, the cells were lysed by ALP lysis buffer (10 mM Tris-HCl, 100 mM NaCl, 1 mM Triton-X 100, 1% PMSF, and 1% protease inhibitor cocktail, pH 7.4) and the lysates were then analyzed by the use of SIGMAFAST™ p-Nitrophenyl phosphate Tablets (Sigma Aldrich, St Louis, MO, USA) according to the manufacturer’s instruction after 15 min. The ALP activity was normalized by the total protein content measured by BCA Protein Assay Kit (Thermo Fisher Scientific, Waltham, MA, USA).

### 4.8. Mineralization Assay

Mineralization of the cells was examined by alizarin red S (Sigma Aldrich, St Louis, MO, USA) staining after 3 weeks. The cells were washed, fixed, then stained with 2% alizarin red S (pH 4.2) for 45 min. Excess dye was removed by washing with distilled water, then alizarin red S were extracted with 10% cetylpyridinium chloride (Sigma Aldrich, St Louis, MO, USA) solution and absorbance was determined at the wavelength of 570 nm.

### 4.9. Statistics

A multiple comparison *t*-test was used to compare the means of different pairs of populations (e.g., two media or cell lines) using the Bonferroni-Dunn method. Data are expressed as mean values ± standard deviation of means (*n* = 3).

## Figures and Tables

**Table 1:** The amount of BMP-7 protein in the supernatants of DPSC-BMP-7 cell line cultured in different media (*n* = 3).

	CM	CM+	OM	OM+
**Day 1**	0.02 ng	17.3 ng	0.09 ng	18.7 ng
**Day 2**	0.23 ng	34.2 ng	0.21 ng	51.7 ng
**Day 3**	0.24 ng	77.3 ng	0.27 ng	105.5 ng

## Data Availability

The data presented in this study are available on request from the corresponding author.
